# Reverse-view dual-traction endoscopic submucosal enucleation of a large gastric cardia gastrointestinal stromal tumor

**DOI:** 10.1055/a-2895-4838

**Published:** 2026-07-08

**Authors:** Shan-Shan Hu, Wei-Hui Liu

**Affiliations:** 1 Department of Gastroenterology and Hepatology, Sichuan Academy of Medical Sciences & Sichuan Provincial People’s Hospital 89669 School of Medicine, University of Electronic Science and Technology of ChinaChengduSichuanChina

Endoscopic resection of large (>3 cm) submucosal tumors (SMTs) at the gastroesophageal junction remains a formidable challenge. The acute angle, narrow lumen, and proximity to the mediastinum render submucosal tunneling resection technically unfeasible and endoscopic full-thickness resection high-risk for perforation. The reverse-view position, conventionally used for gastric fundal procedures, has been underutilized for cardia lesions due to the perceived difficulty in orientation and instrument control. We describe a novel strategy combining reverse-view positioning with dual internal–external traction to achieve pure endoscopic enucleation of a 5-cm gastric cardia gastrointestinal stromal tumor (GIST) 1​ 2​ ​3​ .

**Fig. 1:**
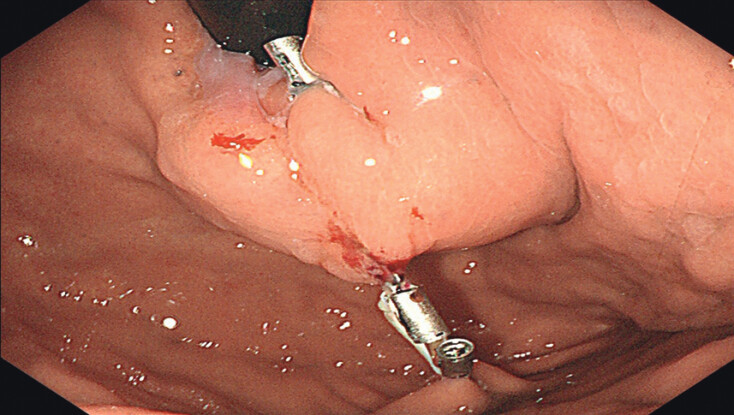
Reverse-view with dual-traction: rubber band and dental floss.

**Fig. 2:**
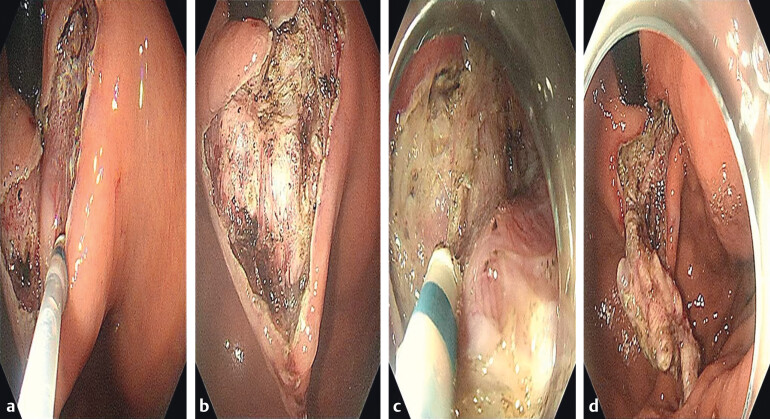
“Peanut-shelling” dissection: mucosal incision ( **a** ), progressive dissection ( **b** and **c** ), tumor enucleated ( **d** ).

**Fig. 3:**
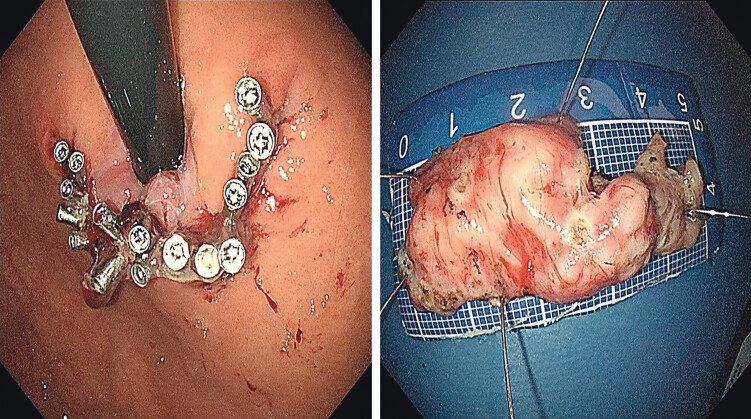
( **a** ) Mucosal flap repositioned and clipped. ( **b** ) The enucleated 5-cm tumor.

A 58-year-old man underwent endoscopic resection of a 5-cm cardia mass (endoscopic ultrasound: muscularis propria origin). The endoscope was retroflexed for reverse-view exposure. Internal rubber-band traction (tumor apex) pulled downward, while external dental-floss traction (fixed to cheeks) provided lateral counter-traction. Gravity synergized with internal traction ( Fig. 1 ). The mucosa was incised and reflected as a flap. Electrosurgical dissection along the muscularis propria plane enucleated the tumor in a “peeling” manner ( Fig. 2 ). The flap was repositioned and secured with hemoclips ( Fig. 3 ). Pathological examination confirmed a GIST with R0 resection. The patient was discharged on day 3. At the 3-month follow-up, the mucosa healed completely without recurrence ( Video 1 ).

**Video 1** Reverse-view dual-traction endoscopic submucosal enucleation of a large gastric cardia gastrointestinal stromal tumor.


Reverse-view dual-traction endoscopic submucosal enucleation integrates three independent forces—gravity, internal rubber-band traction, and external dental-floss counter-traction—to create a self-stabilizing, self-expanding operative field at the gastroesophageal junction. The elastic rubber-band traction conforms to the tumor contour and provides self-adjusting tension, reducing the risk of tissue avulsion compared to rigid traction systems. The mucosal flap technique preserves the mucosal barrier and facilitates wound closure, potentially reducing the risk of postoperative perforation and bleeding. This strategy may enable pure endoscopic resection of large cardia SMTs previously deemed endoscopically unresectable.

Endoscopy_UCTN_Code_TTT_1AO_2AG
